# Sea level rise may increase extinction risk of a saltmarsh ontogenetic habitat specialist

**DOI:** 10.1002/ece3.3291

**Published:** 2017-08-27

**Authors:** David Samuel Johnson, Bethany L. Williams

**Affiliations:** ^1^ Virginia Institute of Marine Science Gloucester Point VA USA

**Keywords:** estuary, extinction risk, gastropods, global change, indirect effects, nursery

## Abstract

Specialist species are more vulnerable to environmental change than generalist species. For species with ontogenetic niche shifts, specialization may occur at a particular life stage making those stages more susceptible to environmental change. In the salt marshes in the northeast U.S., accelerated sea level rise is shifting vegetation patterns from flood‐intolerant species such as *Spartina patens* to the flood‐tolerant *Spartina alterniflora*. We tested the potential impact of this change on the coffee bean snail, *Melampus bidentatus*, a numerically dominant benthic invertebrate with an ontogenetic niche shift. From a survey of eight marshes throughout the northeast U.S., small snails were found primarily in *S. patens* habitats, and large snails were found primarily in stunted *S. alterniflora* habitats. When transplanted into stunted *S. alterniflora*, small snails suffered significantly higher mortality relative to those in *S. patens* habitats; adult snail survivorship was similar between habitats. Because other habitats were not interchangeable with *S. patens* for young snails, these results suggest that *Melampus* is an ontogenetic specialist where young snails are habitat specialists and adult snails are habitat generalists. Temperature was significantly higher and relative humidity significantly lower in stunted *S. alterniflora* than in *S. patens*. These data suggest that thermal and desiccation stress restricted young snails to *S. patens* habitat, which has high stem density and a layer of thatch that protects snails from environmental stress. Other authors predict that if salt marshes in the northeast U.S. are unable to migrate landward, sea level rise will eliminate *S. patens* habitats. We suggest that if a salt marsh loses its *S. patens* habitats, it will also lose its coffee bean snails. Our results demonstrate the need to consider individual life stages when determining a species’ vulnerability to global change.

## 1 INTRODUCTION

The aquatic tadpole becomes the terrestrial toad. The leaf‐eating caterpillar becomes the nectar‐sipping butterfly. Like the toads and the butterflies of the world, more than 80% of animals on Earth exhibit an ontogenetic niche shift (Werner, 1988), though not all require dramatic metamorphosis to do so. In marine ecosystems, for instance, various fishes and invertebrates worldwide have ontogenetic niche shifts in which juveniles rely on estuarine nursery habitats before migrating to oceanic and coastal habitats (Dahlgren & Eggleston, 2000; Beck et al., 2001; Nagelkerken et al., 2002; Nelson et al., 2012). Global change can reduce biodiversity by shrinking or eliminating niches (Brooks et al., 2002; Byrnes, Reynolds, & Stachowicz, 2007; Deegan et al., 2012; Deutsch, Ferrel, Seibel, Pörtner, & Huey, 2015) and as a result, species with ontogenetic niche shifts, which rely on more than one niche throughout their lives, may be particularly susceptible to environmental change.

Empirical and theoretical evidence suggests that specialist species are more sensitive to environmental changes than generalists because generalists are able to take advantage of a wider range of resources along a niche axis (Colles, Liow, & Prinzing, 2009; Devictor, Julliard, & Jiguet, 2008; Fischer & Stöcklin, 1997; Fisher, Blomberg, & Owens, 2003; Goulson, Hanley, Darvill, Ellis, & Knight, 2005; Julliard, Jiguet, & Couvet, 2004; Munday, 2004; Rooney, Wiegmann, Rogers, & Waller, 2004; Warren et al., 2001). For instance, sea level rise may have caused the recent global extinction of the Bramble Cay mosaic‐tailed rat, *Melomys rubicola*, a mammal that specialized on low‐lying coastal habitats (Waller et al., 2017). A specialist can be defined as a species that uses a limited set of noninterchangeable resources, versus a generalist which uses a broad set of interchangeable resources (Rudolf & Lafferty, 2011). Analogous to the species‐level specialists, Rudolf and Lafferty (2011), through mathematical modeling, suggest that ontogenetic specialists (species in which at least one life stage requires a specific, irreplaceable niche) are more vulnerable to environmental change than ontogenetic generalists (each life stage can use a range of niches). Therefore, it may be critical to include complex life cycles in our understanding of the effect of global change on local and regional biodiversity (Nakazawa, 2011; Rudolf & Rasmussen, 2013).

Salt marshes are highly productive, intertidal grasslands found in temperate latitudes and are excellent ecosystems to examine the effects of global change on animals. These ecosystems are replete with species with ontogenetic niche shifts (Alcaraz & Garcia‐Berthou, 2007; Johnson, Fleeger, Galván, & Moser, 2007; Kneib, 2000; Smith, Taghon, & Able, 2000) and are threatened by multiple stressors that reduce habitat niches (Deegan et al., 2012; Morris, Sundberg, & Hopkinson, 2013). In the northeast U.S., climate‐accelerated sea level rise is reducing habitat niches in the high marsh (an area of the marsh irregularly flooded) as flood‐intolerant plants such as *Spartina patens* are being replaced by flood‐tolerant plants such as *Spartina alterniflora* (Warren & Niering, 1993; Carey, Raposa, Wigand, & Warren, 2017; Feagin, Martinez, Mendoza‐Gonzalez, & Costanza, 2010; Morris et al., 2013; Raposa, Weber, Ekberg, & Ferguson, 2017). These changes in vegetated habitats are predicted to lead to secondary extinctions. For instance, Correll et al. (2017) predict the global collapse of the saltmarsh sparrow, *Ammodramus caudatus*, a salt marsh specialist, within the next half‐century, in part, because of sea level driven losses of *S. patens* habitats.

The coffee bean snail, *Melampus bidentatus* (hereafter *Melampus*), is an abundant pulmonate (air‐breathing) gastropod in the salt marshes of the northwest Atlantic and northern Gulf of Mexico (Dennis & Hellberg, 2010). *Melampus* have aquatic, veliger larvae (Apley, 1970) and benthic, semiterrestrial juveniles and adults. In a salt marsh in Connecticut, Fell, Murphy, Peck, and Recchia (1991) found that smaller, younger snails dominate flood‐intolerant *S. patens* habitats and larger, older snails dominate the flood‐tolerant stunted *S. alterniflora* habitats. This suggests an ontogenetic habitat shift occurs for *Melampus* not just from the larvae–to–juvenile stage (Helvenston et al., 1995), but also from the juvenile–to–adult stage. The robustness of this distributional size pattern of snails, however, has not been tested across a large spatial scale.

Here, we examine how predicted changes in saltmarsh vegetation patterns induced by sea level rise may affect the population of the coffee bean snail. First, we conducted a survey of salt marshes across the northeast U.S. to test the generality of the size distribution pattern (i.e., are small snails typically found in *S. patens* and large ones in stunted *S. alterniflora*?). Second, we tested the hypothesis that *Melampus* is an ontogenetic specialist with small, young snails specializing on the *S. patens* habitat before shifting to stunted *S. alterniflora* as large adults. A key characteristic of an ontogenetic specialist is that for at least one life stage, other niches (in this case, habitats) are not interchangeable (Rudolf & Lafferty, 2011). Therefore, we tested the survivorship of each size class in multiple habitats. If *Melampus* is an ontogenetic habitat specialist at the young age class, then survivorship of young snails should be greater in *S. patens* habitats than in other habitats.

## 2 METHODS

### 2.1 General site description

**Figure 1:**
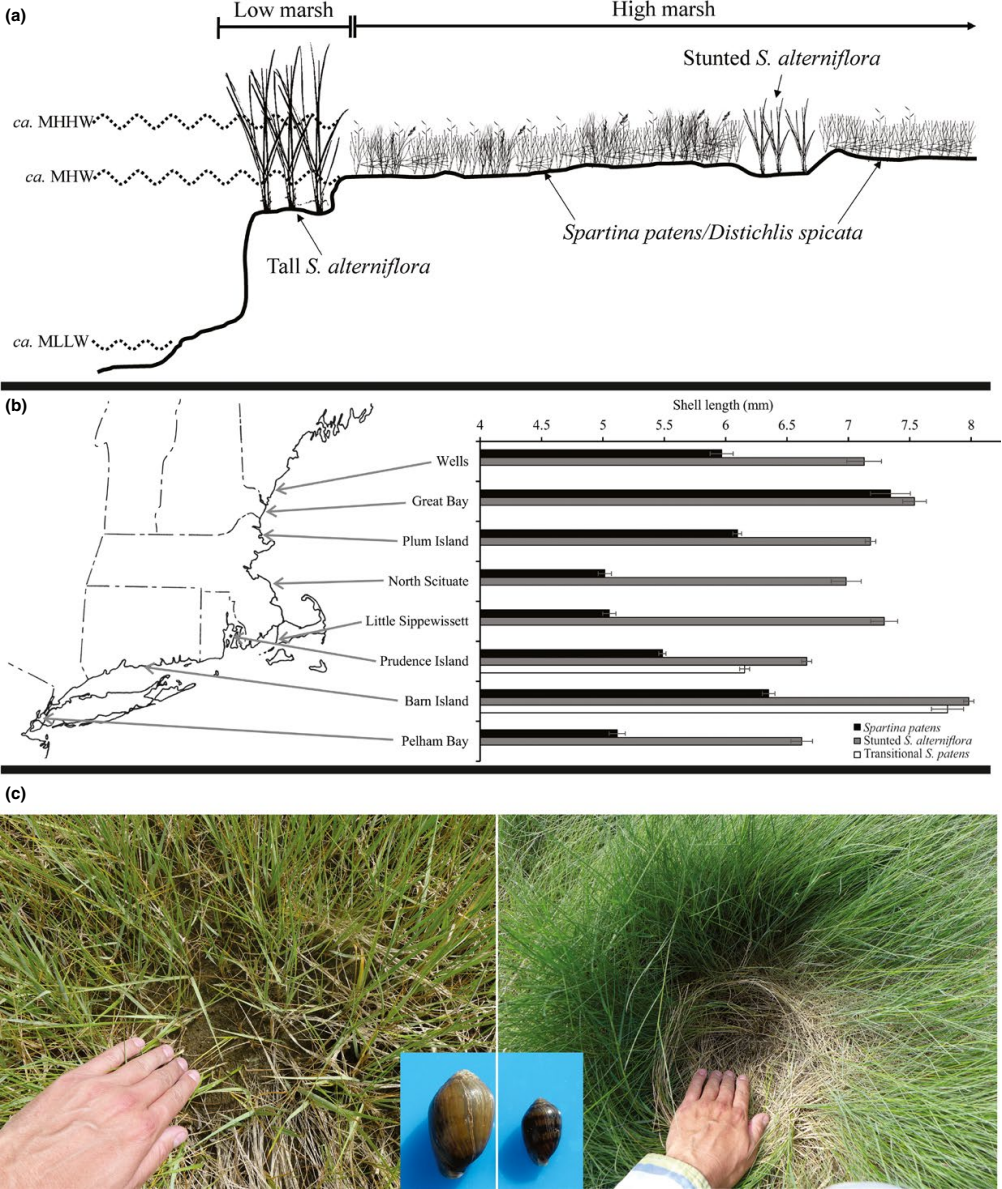
(a) Generalized cross‐section of salt marshes in the northeast United States showing major vegetation patterns. MLLW, mean low low water; MHW, mean high water; MHHW, mean high high water (e.g., spring tides). (b) Map of sites with snail size data. Data are mean (±1 *SE*) shell length of snails collected in different habitats from eight salt marshes in the northeast United States. Transitional *Spartina patens* only found in Prudence Island and Barn Island. Data for tall *Spartina alterniflora* are not displayed because only two snails were found in this habitat (11.3 and 11.0 mm shell length) and only in the Pelham Bay site. (c) A comparison of the relative size of snails from two different marsh habitats from the marshes of the Plum Island Estuary. Snails are larger in the stunted *S. alterniflora* (left panel), which has recurrent exposed ground due to little thatch and canopy cover, than in the *S. patens* habitat (right panel), which has high stem density and a thick thatch layer. Note the dark banding on the smaller snail, that is prevalent on young and juvenile snails but grows faint in large adults. While snails are scaled appropriately to each other, they are enlarged to show detail and therefore not to scale of the habitats. Photograph credits: David Samuel Johnson

In the northwest Atlantic, salt marshes are a mosaic of habitats including tidal creeks, vegetated habitats, and shallow ponds. In terms of vegetation patterns, tall *S. alterniflora* (smooth cordgrass) forms a monoculture belt in the regularly flooded (i.e., daily) intertidal low marsh; whereas the irregularly flooded high marsh is a mosaic of habitats dominated by *S. patens* (salt hay) and *Distichlis spicata* (spikegrass) (hereafter referred to as *S. patens* habitats) interspersed with water‐logged pannes of stunted *S. alterniflora* (Miller & Egler, 1950; Niering & Warren, 1980 Fig. 1a). *Spartina patens* habitats have high stem densities with standing dead stems from previous years forming mats (thatch) (Johnson 2011, Johnson et al. 2016). *Spartina alterniflora* habitats have lower stem densities with little to no thatch layer (Fig. 1c).

### 2.2 Regional survey

While the literature suggests that small *Melampus* are the dominant size class in *S. patens* habitats, large snails are the dominant size class in stunted *S. alterniflora* habitats (Fell et al., 1991), and individuals of either size class are rarely found in the low marsh (Fell et al., 1982; Vince, Valiela, Backus, & Teal, 1976), this has not been tested across broad spatial scales. To determine if this is a general pattern, we surveyed eight marshes along the northeastern U.S. (Fig. 1, Table S1). At each site ten 0.0625 m^2^ quadrats were haphazardly tossed in each of the three studied habitats: tall *S. alterniflora*, stunted *S. alterniflora*, and *S. patens*. The tall *S. alterniflora* habitats were limited (<0.25 m wide) in the North Scituate site and not surveyed. At the Great Bay, New Hampshire, and Little Sippewissett, Massachusetts sites, the stunted *S. alterniflora* habitats were not in isolated pannes but were instead adjacent to the tall *S. alterniflora* habitat. *Melampus* and all other invertebrates were enumerated in each quadrat. Shell length was measured for all *Melampus* collected using digital calipers. To examine the relationship between snail size and environmental stress, humidity and temperature at ground (snail) level was measured in each quadrat with a digital hygrometer/thermometer. Stem densities for tall *S. alterniflora* were estimated by counting all stems within the entire quadrat. Because stem densities are higher in *S. patens* and stunted *S. alterniflora* than tall *S. alterniflora* we followed Johnson, Warren, Deegan, and Mozdzer (2016) and estimated stem densities for these species in a 72.25 cm^2^ quadrat placed adjacent to the larger quadrat.

At the Barn Island, Connecticut, and Narragansett Bay, Rhode Island, marshes we observed large patches of *S. patens* with shorter stems and no thatch layer (the bare substrate easily observed from above; DS Johnson, BL Williams, personal observation). *Spartina patens* is a flood‐intolerant species, and as sea level rises and flooding increases plant growth is limited until the habitat switches to the flood‐tolerant stunted *S. alterniflora* habitat. Thus, these areas were transitional areas from *S. patens* to *S. alterniflora* as sea level rise increased flooding stress (Carey et al., 2017; Raposa et al., 2017, hereafter referred to as transitional *S. patens*). To compare these transitional habitats with established habitats, we sampled ten additional plots in these transitional areas following the methods described above for *S. patens*. Given the low stem density and lack of thatch, we hypothesized that snails in these transitional habitats would be larger than those in the *S. patens* habitats.

#### 2.2.1 Statistical analyzes

All statistical analyzes in this study were conducted in R (version 3.0.3, R Core Team, 2014). To test the effect of habitat (*S. patens*, transitional *S. patens*, and stunted *S. alterniflora*) on snail length, temperature, and humidity, data for each response variable were analyzed separately with a linear mixed effect model using the lme function in the nlme package in R (Pinherio et al., 2016). Only two snails were found in the tall *S. alterniflora* habitats throughout the region and were excluded from analyzes. Habitat was considered a fixed effect. Site was considered a random effect because our goal was not to test the effect of site on response variables, but to control for the natural variability among sites while testing the effect of habitat on snail size. Snail length was averaged per plot (thus, *n* = 10/habitat) prior to analysis.

### 2.3 Experiments

To test the hypothesis that *Melampus* is an ontogenetic specialist and that tall and stunted *S. alterniflora* habitats are not interchangeable with *S. patens* for young snails we conducted two manipulative experiments, both of which were completed in the Plum Island Estuary, Massachusetts. For these experiments we focused on two size classes of snails, large mature adults (8.5–11.0 mm shell length, hereafter “adult snails”) and juveniles/young adults (4.5–7.0 mm shell length, hereafter “young snails”). *Melampus* are typically sexually mature by 6 mm shell length (Apley, 1970); thus, our “young” age class may include young, egg‐laying adults. Because our hypotheses are based on the suitability of habitats for different size classes of snails, we binned snails into size classes that corresponds to sizes typically found in the *S. patens* and stunted *S. alterniflora* habitat previously observed in Plum Island marshes (Johnson, 2011). As a result, we consider ontogeny based not necessarily on life stage (i.e., sexually immature vs. sexually mature), but on a size class associated with a particular habitat.

#### 2.3.1 Environmental stress experiment

To test the substitutability of habitats based on environmental stressors (i.e., temperature, humidity, and inundation), a reciprocal transplant experiment was performed. Ten snails of each age class were caged together in each of the three habitats (*n* = 10 cages per habitat): tall *S. alterniflora*, stunted *S. alterniflora*, and *S. patens*. Young snails were collected from the *S. patens* habitats and adult snails from stunted *S. alterniflora* habitats. Each cage was constructed of polyvinyl chloride pipe with tear resistant 1‐mm size mesh covering all sides and the top. Cages were 25 cm long, 25 cm wide, and 25 cm tall and were dug into the ground 5 cm to prevent snails from escaping. Experimental snails were measured for shell length and painted with white permanent marker to differentiate them from snails already in cage areas (no attempt was made to remove snails already in these plots). At the beginning of the experiment we took temperature and humidity measurements to test for potential caging effects. Cages were inspected 2–3 times per week, and the area surrounding the cages was searched for potential escapees. Cages were deployed 23 June 2014 and retrieved 41 days later on 4 August 2014.

To estimate survivorship after the experiment, all experimental *Melampus* were set into trays. Once they moved on their own, they were counted as live. After thirty minutes, if any *Melampus* had not moved, they were flooded with seawater. If they made no movement after another 30 min, they were counted as dead. Not all experimental *Melampus* were recovered. This may be because some snails died early in the experiment and their empty shells, which become brittle and easily abraded when empty, could not be identified because tidal action abraded the paint marker and/or destroyed them. Alternatively, these missing snails may have escaped the cages, although we did not find any painted snails outside the cages during our periodic maintenance checks. Because we cannot definitively determine the fate of the missing snails these were excluded from our analyzes.

##### 2.3.1.1 Statistical analyzes

Because there was variance in the number of snails missing among cages, we standardized the data with a proportional weighting factor. In each replicate cage, the number of surviving snails was multiplied by the following weighting factor, *W*_*i*_:

$$W_{i}\;=\;n_{i}/N_{i}$$

where *n *= the number of snails retrieved and *N *= the number of snails placed in the cage at the beginning of the experiment. This procedure downweighted samples with a higher proportion of missing snails, promoting inferences from samples with greater representation. The weighted data were then analyzed with a one‐way ANOVA (age classes analyzed separately) to compare habitat differences in survivorship (aov function). Tukey's Honest Significant Difference tests were used for pairwise comparisons if main effects were significant.

#### 2.3.2 Predation experiment

To test the substitutability of habitats based on predation pressure, a tethering experiment was performed in three habitats (tall *S. alterniflora*, stunted *S. alterniflora*, and *S. patens*) at the Plum Island site. Snails were tethered to a plastic flag using clear fishing line glued to top of their shells. Ten snails of each age class were placed haphazardly in each of the three habitats. After 24 hr, tethers were collected. Snails with cracked shells, missing, or with only a piece of shell attached to the tether were scored as consumed. We are confident that missing snails can be counted as consumed and not escapees because of the strength of the tethers and the short duration of the experiments. This experiment was performed twice. Once during a mid tide, which floods only the tall *S. alterniflora* and once during a spring tide, which floods the high marsh (stunted *S. alterniflora* and *S. patens*).

##### 2.3.2.1 Statistical analyzes

We tested the effect of habitat, tide type, and age class on snail survival by fitting the data to a binomial logistic regression model with logit link and then conducting an analysis of deviance.

### 2.4 Stress‐tolerance study

On humid, cool mornings during the summer, *Melampus* can be seen in open habitats (such as footpaths and areas denuded by wrack), where they may be attracted to high algal biomass (Pascal & Fleeger, 2013; Thompson, 1984). By midday, if the temperatures are high and the humidity is low (e.g., a clear sunny day in July), *Melampus* retreat into grass. To determine how environmental stress such as temperature and humidity can influence size class distributions and daily movements we conducted a study of these diurnal movements. To photo‐document these diurnal movements we set up a game camera (Bushnell Trophy Camera, Model 119436) with an infrared flash (for nighttime images) in abandoned footpaths adjacent to *S. patens* habitats. It was set to capture images at 5‐min intervals over 3 days. From these images we observed that snails flooded into the footpath at night and ebbed back into the grass by midday (https://www.youtube.com/watch?v=mmjMf_SsktQ).

To examine at the potential relationship between temperature/humidity and snail habitat choice, we set up ten 0.04 m^2^ plots along an abandoned path denuded of vegetation on 30 June 2014. Each plot was marked by string tied to bamboo staked at the four corners. Beginning at 10:00, snails in each plot were enumerated, and humidity and temperature were measured hourly for 24 hr with a digital hygrometer/thermometer. A digital picture of each plot was taken from the same height every hour. These pictures were used for density estimates and length measurements using imageJ (Schneider, Rasband, and Eliceiri, 2012) software (calibrated to the premeasured size of the hygrometer in each plot). This process was repeated every hour for 24 hr, except at hours 03:00 and 04:00, when the high marsh was flooded.

#### 2.4.1 Statistical analyzes

To determine if adult snails responded differently than young snails to changes in temperature and relative humidity in terms of density, we ran an analysis of covariance (ANCOVA) on log‐transformed data. Temperature and humidity were analyzed separately. There was no effect of size class on density for neither humidity nor temperature (*p* ≥ .33). Densities were pooled, and simple linear regression models were employed to examine the relationship between snail density and temperature and humidity separately.

## 3 RESULTS

### 3.1 Regional survey

In our survey that extended along 600 km of coastline in the U.S. northeast from New York to Maine, habitat had a significant effect on snail size (*p* < .001). Based on regional means (i.e., mean of data pooled across sites) of snail size, individuals were smallest in the *S. patens* habitats (5.6 ± 0.03 mm, mean ± *SE*), largest in stunted *S. alterniflora* (7.2 ± 0.02 mm shell length) with snails in transitional *S. patens* habitats falling in between (6.3 ± 0.05 mm). The only two snails found in the tall *S. alterniflora* habitats in the region were located in one plot in New York (11.3 and 11.0 mm). Regionally, snail density was on average highest in *S. patens* (595 ± 44 snails per m^2^), with densities higher in stunted *S. alterniflora* (464 ± 47 m^−2^) than in transitional *S. patens* (465 ± 100 snails per m^2^) (Table S3).

**Figure 2:**
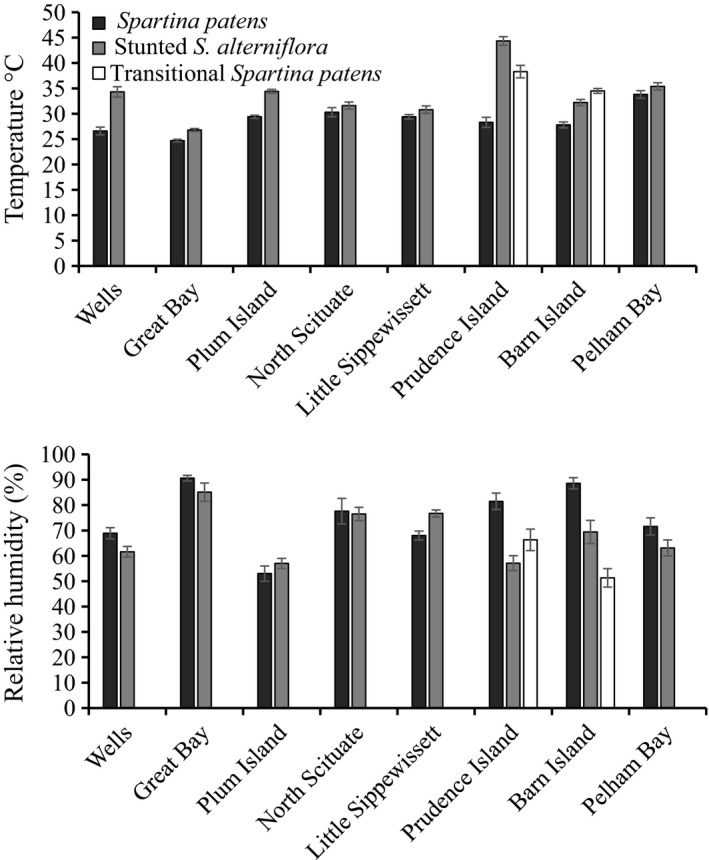
Mean (±1 *SE*) relative humidity and temperature at sediment surface beneath *Spartina patens* and stunted *Spartina alterniflora* canopies from eight sites in the northeast US

Habitat significantly influenced air temperature and relative humidity beneath the grass canopy (*p* < 0.001). Regional mean temperature was 4.8°C warmer in stunted *S. alterniflora* (33.6°C) than in transitional *S. patens* (36.4°C) and 7.7°C warmer than in *S. patens* (28.8°C) (Fig. 2, Table S4). Regionally, relative humidity was higher, on average, in *S. patens* (75%) than in transitional *S. patens* (59%) and stunted *S. alterniflora* (68%) (Fig. 2, Table S5).

Regional mean stem densities were 18,860 (±1,398) stems per m^2^ for *S. patens*, 8,202 (±1,644) stems per m^2^ for transitional *S. patens*, 578 (±90) stems per m^2^ for tall *S. alterniflora*, and 1,800 (±160) stems per m^2^ for stunted *S. alterniflora* (Table S6).

### 3.2 Environmental stress experiment

**Figure 3:**
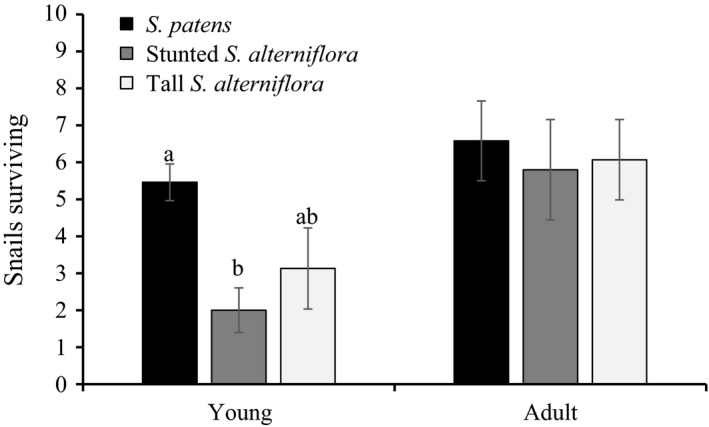
Weighted mean (±1 *SE*) survivorship of *Melampus* from transplant studies. Based on a one‐way analysis of variance, habitat had a significant effect on young snails (*p* = .009) but not adult snails (*p* = .89). Bars that share a letter are not significantly different based on Tukey's honest significant difference test

Habitat affected the survivorship of young snails (ANOVA, *F* = 5.705, num *df* = 2, den def = 26, *p* = .009), with 64% higher survivorship in the *S. patens* habitats (weighted mean of 5.5 snails) than in the stunted *S. atlerniflora* (weighted mean of 2 snails, Tukey's *p* = .007, Fig. 3). Although survivorship of young snails was lower in tall *S. alterniflora* habitats (weighted mean of 3.1 snails) than *S. patens* habitats, it was not significantly so (Tukey's *p* = .10). This suggests that, at least for the duration of our experiment, young *Melampus* are not susceptible to the higher inundation stress of the low marsh. Habitat did not influence adult snail survivorship (ANOVA, *F* = 0.115, num *df* = 2, den df = 26, *p* = .89) with a weighted mean survivorship of 5.5–6.5 snails (Fig. 3).

### 3.3 Predation experiment

**Figure 4:**
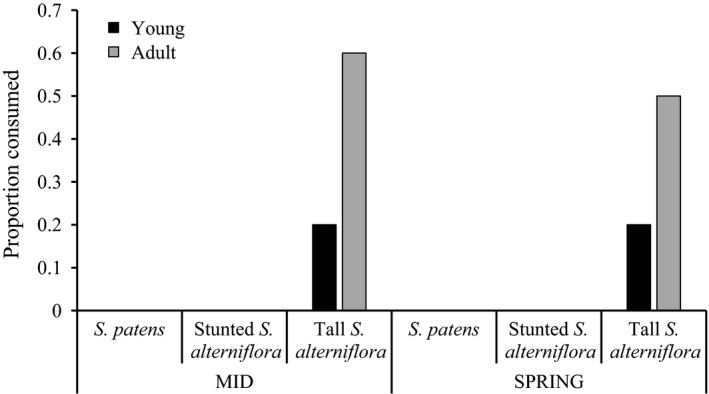
Significant effect of habitat (logistic regression, *p* < .001) and age class (logistic regression, *p* = .02) on the proportion of snails consumed from 24‐hr predation experiments conducted in the marshes of the Plum Island Estuary, Massachusetts. MID, middle tides that did not flood the high marsh; SPRING, spring tides that flooded the high marsh. Tide type (mid tides did not flood the high marsh, spring tides did) had no effect on snail mortality (logistic regression, *p* > .05)

*Melampus* of both age classes experienced significantly higher mortality in the low marsh, (i.e., tall *S. alterniflora*, 20%–60%) than the high marsh habitats (0%) after 24 hr of exposure to predator access (logistic regression, *p* < .001), regardless of tide type (logistic regression, *p* > .05) (Fig. 4). The presence of cracked shells suggests that crabs were predators in addition to fish. Adult snails had significantly higher predation rates (50%–60%) than the smaller, young snails (20%) (logistic regression, *p* = .02).

### 3.4 Stress‐tolerance study

**Figure 5:**
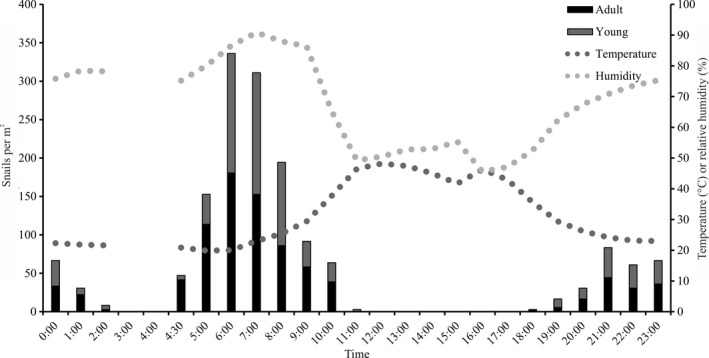
Significant relationship between mean *Melampus* density and (a) temperature (Linear regression: *R* ^2^ = 0.32, *p* = .005) and (b) relative humidity (Linear regression: *R* ^2^ = 0.57, *p* < .001) in open habitats over 24 hr. Gap in temperature and humidity lines due to level of water in plot at 3:00 and 4:00, which did not allow us to make measurements

Near‐surface air temperature had a significant negative relationship with *Melampus* density (regression test *p* = .005, *R* ^2^ = 0.32; Fig. 5). However, there was a threshold at 46°C, above which, no *Melampus* were found in these open areas. Humidity had a significant positive relationship with *Melampus* density (regression test *p* < .001, *R* ^2^ = 0.57; Fig. 5). A humidity threshold existed at 55%, below which no snails were found in the open habitats. Because temperature and humidity co‐vary, we cannot tease apart these effects on snail density and movement.

## 4 DISCUSSION

Ontogenetic specialists may be susceptible to climate change impacts because of a high degree of specialization at a particular life stage. Our data suggest that *Melampus* is an ontogenetic habitat specialist in which young snails are habitat specialists and adult snails are habitat generalists. Specifically, young snails specialize on *S. patens* habitats, which facilitates young snail survivorship by reducing environmental stress and predation pressure. If the high marsh is unable to migrate landward (Kirwan, Temmerman, Skeehan, Guntenspergen, & Fagherazzi, 2016; Smith, 2013), models predict that salt marshes in the U.S. east and Gulf coasts will lose their high‐marsh habitats and become monocultures of *S. alterniflora* by 2100 (Feagin et al., 2010; Morris et al., 2013). Other authors suggest that when a salt marsh loses it *S. patens* habitats, it will lose its charismatic and vulnerable species such as saltmarsh sparrows, *Ammodramus caudacutus*, (DiQuinzio, Paton, & Eddleman, 2001; Gjerdrum, Elphick, & Rubega, 2005, Correll et al., 2017). We suggest it will also lose its coffee bean snails.

Fell et al. (1991) found that *Melampus* were consistently smaller in the *S. patens* habitats versus stunted *S. alterniflora* habitats in a Connecticut salt marsh. We found a similar pattern in the eight salt marshes from New York to Maine. We hypothesize that this spatial pattern is the result of the lower thermal/desiccation tolerance limits of smaller snails (stress‐tolerance hypothesis, Price, 1980, 1984). Temperature was consistently higher and humidity consistently lower in the stunted *S. alterniflora* habitats than in *S. patens* habitats (Tables S4 and S5), which reduced survivorship of young snails by 64% in stunted *S. alterniflora*. The significant relationship of snail density with temperature (negative) and humidity (positive) from our stress‐tolerance study implicates the role of environmental stress in controlling snail habitat selection.

Our hypothesis that large snails are able to withstand higher stress, and therefore, use multiple habitats are also supported by data from the transitional *S. patens* habitats found in Barn Island, Connecticut and Prudence Island, Rhode Island. Snails in these habitats were larger than those in typical *S. patens* habitats. Vast expanses of *S. patens* are being replaced by stunted *S. alterniflora* in these marshes via greater flooding stress due to sea level rise (Niering & Warren, 1980; Raposa et al., 2017). As flood‐intolerant *S. patens* experiences greater flooding stress, stem density and shoot height decreases and the thatch layer is lost (Carey et al., 2017). As a result, ground (snail‐level) temperature increases and humidity decreases and these transitional habitats are more similar to stunted *S. alterniflora* habitats than *S. patens* in terms of environmental conditions, which are also reflected in the size structure of snails. This emphasizes the importance of the thatch layer of *S. patens* habitats in protecting young snails from solar radiation and low humidity (Price, 1980).

Food limitation is an alternative hypothesis to explain why snails in *S. patens* were smaller than those in stunted *S. alterniflora* (Zajac, Kelly, Perry, & Espinosa, 2017). While *Melampus* is considered a detritivore (Thompson, 1984) it also consumes benthic algae, a higher quality food than plant detritus (Pascal & Fleeger, 2013). Benthic microalgae were twice as abundant in stunted *S. alterniflora* habitats than in *S. patens* habitats (Fig. S1). We do not think food limitation is driving the size patterns because our stress‐tolerance study found that snails move freely from algae‐limited vegetated habitats and algae‐rich unvegetated habitats when humidity is high and temperatures are low. Although not specifically tested here, this suggests that small snails may move from *S. patens* to stunted *S. alterniflora* to forage when environmental stress is low. Food availability, however, may explain the ontogenetic habitat a shift from *S. patens* to stunted *S. alterniflora*. *Spartina patens* provides food‐poor, but less stressful habitat than stunted *S. alterniflora*, which provides a food‐rich but more stressful habitat (Fig. S1). Thus, *Melampus* may leave *S. patens* for a more profitable foraging habitat when they are large enough to tolerate the higher temperature and lower humidity of stunted *S. alterniflora*.

In a scenario where the infrequently flooded high marsh converts into the daily flooded low marsh as a result of sea level rise (Feagin et al., 2010; Morris et al., 2013), our results suggest that *Melampus* would have high mortality because of predation pressure, not inundation stress. Despite being an air‐breathing snail, *Melampus* of both age classes tolerated the high inundation stress associated with life in the low marsh, at least for the duration of our caging experiments (41 days). However, in our predation experiments, ~60% of the adults and 20% of the young snails were consumed in the low marsh; whereas 100% of the snails in both age classes survived in the high‐marsh habitats regardless of tide type (mid vs. spring tides). The differential response of each snail age class to predation is likely the result of optimal foraging by green crabs, *Carcinus maenas*, that choose more energetically profitable prey (large snails) over less profitable ones (small snails) (Lawton & Hughes, 1985).

The term “specialist” is often applied at the species level in which the species utilize a single resource (food, habitat). But an organism can be a specialist at one life stage but not another (Rudolf & Lafferty, 2011). For instance, *Melampus* has long been considered a generalist species (Hausman, 1932) because it has a broad diet (Thompson, 1984) and occupies multiple habitats (Fell et al., 1982; Johnson, 2011; Price, 1980; Russell‐Hunter, Apley, & Hunter, 1972; Spelke, Fell, & Helvenston, 1995; Vince et al., 1976). We demonstrate, however, that habitats other than *S. patens* are not interchangeable for young age classes in the U.S. northeast and *Melampus* may be an ontogenetic habitat specialist. The distinction between a whole‐species specialist and an ontogenetic specialist is important to predict the response of natural communities to climate change.

Climate change can lower biodiversity by accelerating extinctions due to direct effects on a species’ physiology (e.g., thermal tolerance) (Sinervo et al., 2010; Thomas et al., 2004; Urban, 2015). We show that it may also threaten biodiversity and accelerate extinctions indirectly by eliminating or shrinking niches such as food or habitat availability. A species’ vulnerability to extinction depends on its level of niche specialization (i.e., niche width). In species with ontogenetic niche shifts, a greater degree of niche specialization at one life stage can confer a greater risk of extinction due to a loss in niche heterogeneity. As we show here, ontogenetic niche specialists may be particularly susceptible to extinction due to climate change. Our results thus underscore the need for future researchers to consider a species’ ecology, in addition to its physiology, when determining its risk of extinction in the face of climate change.

## AUTHORS’ CONTRIBUTIONS

DSJ designed experiments, collected samples, analyzed data, and wrote the first draft. BW conducted experiments, collected and analyzed data, and contributed significantly to revisions. Both authors contributed significantly to the drafts and approved the final version for publication.

## CONFLICT OF INTEREST

We declare no conflict of interests.

## DATA ACCESSIBILITY

Data will be archived at the Plum Island Ecosystem Long Term Ecological Research site (http://pie-lter.ecosystems.mbl.edu/), which is publically available.
