# Supplementary material for: Sea level rise may increase extinction risk of a saltmarsh ontogenetic habitat specialist
Source: Ecol Evol. 2017 Aug 27;7(19):7786–95. doi: 10.1002/ece3.3291 (PMC5632627; doi:10.1002/ece3.3291)
Supplement: Supplementary file 1 [file ECE3-7-7786-s001.docx]

Table S1: Sites selected for surveys.

| Label | City, State | Location | Marsh Name | Latitude, Longitude | Notes |
| --- | --- | --- | --- | --- | --- |
| Wells | Wells, Maine | Rachel Carson National Wildlife Refuge | Depot Brook | 43.320278 N  -70.566667 W | National Estuarine Research Reserve |
| Great Bay | Greenland, New Hampshire |  | Great Bay | 43.134444 N  -70.549722 W | National Estuarine Research Reserve |
| Plum Island | Rowley, Massachusetts | Plum Island Estuary | West Creek | 42.739853 N  -70.849269 W | Long-Term Ecological Research Site |
| North Scituate | North Scituate, Massachusetts |  | Musquashcut Brook | 42.22611 N  -70.774167 W |  |
| Little Sippewissett | Falmouth, Massachusetts |  | Little Sippewissett | 41.576944 N  -70.638611 W |  |
| Prudence Island | Prudence Island, Rhode Island | Narragansett Bay | Nag | 41.623889 N  -70.325000 W | National Estuarine Research Reserve |
| Barn Island | Stonington, Connecticut | Barn Island | Barn Island | 41.338611 N  -70.865556 W |  |
| Pelham Bay | Pelham (New York City), New York | Pelham Bay |  | 40.870864 N  -70.849269 W |  |

Table S2: Mean±standard error (*n* in parentheses) of snail length (mm) from three habitats in eight marshes in the northeast United States. Data from tall *S. alterniflora* not provided in the table as only two snails found in this habitat throughout the region (11.3 and 11.0 mm shell length). Regional values are based data pooled from all marshes.

| Location | *S. patens* | Stunted *S. alterniflora* | Transitional *S. patens* |
| --- | --- | --- | --- |
| Wells | 6.0±0.4 (10) | 7.1±0.4 (10) |  |
| Great Bay | 7.3±0.9 (10) | 7.5±0.7 (10) |  |
| Plum Island Estuary | 6.1±0.3 (10) | 7.2±0.3 (10) |  |
| North Scituate | 5.0±0.4 (10) | 7.0±0.9 (10) |  |
| Little Sippewissett | 5.1±0.3 (10) | 7.3±0.6 (10) |  |
| Narragansett Bay | 5.5±0.3 (10) | 6.7±0.3 (10) | 6.2±0.3 (10) |
| Barn Island | 6.4±0.4 (10) | 8.0±0.3 (10) | 7.8±0.5 (10) |
| Pelham Bay | 5.1±0.5 (10) | 6.6±0.5 (10) |  |
|  |  |  |  |
| Regional | 5.6±0.02 (80) | 7.2±0.03 (80) | 6.6±0.05 (20) |

Table S3: Mean±standard error (*n* in parentheses) of snail density (# m^-2^) from eight marshes in the northeast United States.

| Location | *S. patens* | Stunted *S. alterniflora* | Transitional *S. patens* |
| --- | --- | --- | --- |
| Wells | 266±86 (10) | 142±41 (10) |  |
| Great Bay | 75±72 (10) | 219±40 (10) |  |
| Plum Island Estuary | 843±96 (10) | 1016±181 (10) |  |
| North Scituate | 623±185 (10) | 104±72 (10) |  |
| Little Sippewissett | 562±255 (10) | 101±46 (10) |  |
| Narragansett Bay | 1202±160 (10) | 755±117 (10) | 752±140 (10) |
| Barn Island | 792±116 (10) | 709±91 (10) | 178±67 (10) |
| Pelham Bay | 1003±103 (10) | 430±87 (10) |  |
|  |  |  |  |
| Regional | 595±44 (80) | 464±47 (80) | 465±100 (20) |

Table S4: Mean±standard error (*n* in parentheses) of temperature (°C) taken at ground level beneath the grass canopy from eight marshes in the northeast United States. Regional values are based data pooled from all marshes.

| Location | *S. patens* | Stunted *S. alterniflora* | Transitional *S. patens* |
| --- | --- | --- | --- |
| Wells | 26.6±0.8 (10) | 34.3±1.0 (10) |  |
| Great Bay | 24.7±0.3 (10) | 26.8±0.3 (10) |  |
| Plum Island Estuary | 29.4±0.3 (10) | 34.4±0.4 (10) |  |
| North Scituate | 30.3±0.9 (10) | 31.6±0.7 (10) |  |
| Little Sippewissett | 29.4±0.4 (10) | 30.8±0.7 (10) |  |
| Narragansett Bay | 28.3±1.0 (10) | 44.3±0.8 (10) | 38.3±1.2 (10) |
| Barn Island | 27.8±0.6 (10) | 32.2±0.6 (10) | 34.5±0.5 (10) |
| Pelham Bay | 33.8±0.7 (10) | 35.4±0.7 (10) |  |
| Regional | 28.8±0.4 (80) | 33.6±0.6 (79) | 36.4±0.8 (20) |

Table S5: Mean±standard error (*n* in parentheses) of relative humidity (%) taken at ground level beneath the grass canopy from eight marshes in the northeast United States. Regional values are based data pooled from all marshes.

| Location | *S. patens* | Stunted *S. alterniflora* | Transitional *S. patens* |
| --- | --- | --- | --- |
| Wells | 69±2 (10) | 62±2 (10) |  |
| Great Bay | 91±1 (10) | 85±4 (10) |  |
| Plum Island Estuary | 53±3 (10) | 57±2 (10) |  |
| North Scituate | 78±5 (10) | 77±3 (10) |  |
| Little Sippewissett | 68±2 (10) | 77±1 (10) |  |
| Narragansett Bay | 82±3 (10) | 57±3 (10) | 66±4 (10) |
| Barn Island | 89±2 (10) | 69±5 (10) | 51±4 (10) |
| Pelham Bay | 72±3 (10) | 63±3 (10) |  |
|  |  |  |  |
| Regional | 75±2 (80) | 68±1 (79) | 59±3 (20) |

Table S6: Mean±standard error (*n* in parentheses) of stem density (# m^-2^) from eight marshes in the northeast United States.

| Location | *S. patens* | Stunted *S. alterniflora* | Transitional *S. patens* | tall *S. alterniflora* |
| --- | --- | --- | --- | --- |
| Wells | 21,841±2213 (10) | 3848±355 (10) |  | 1772±242 (10) |
| Great Bay | 16,861±2312 (10) | 1080±74 (10) |  | 1772±242 (10) |
| Plum Island Estuary | 24,734±2739 (10) | 2865±527 (10) |  | 222±9 (58) |
| North Scituate | 23,225±3327 (10) | 1827±237 (10) |  | NA |
| Little Sippewissett | 2816±170 (10) | 395±35 (10) |  | 390±61 (5) |
| Narragansett Bay | 32,567±2460 (10) | 2547±285 (10) | 1995±215 (10) | 2007±494 (10) |
| Barn Island | 27,336±2310 (10) | 1633±182 (10) | 14,408±1674 (10) | 4471±490 (10) |
| Pelham Bay | 1698±189 (10) | 206±27 (10) |  | 2228±230 (10) |
|  |  |  |  |  |
| Regional | 18,860±1398 (80) | 1800±160 (80) | 8202±1644 (20) | 578±90 (55) |

NA=data not available

Figure S1: Mean (±1 SE) sediment chlorophyll *a* (mg m^-2^) for *Spartina patens* (SP), stunted *S. alterniflora* (SSA), and tall *Spartina alterniflora* (TSA) habitats in the Plum Island Estuary. Values averaged across years 2003-2011 based on data collected by the Plum Island Estuary-Long Term Ecological Research program (n=183-468). Different letters indicate significant differences in chlorophyll *a* among habitats (Tukey’s honest significant difference tests).
